# Trigonelline as an anticonvulsant agent: mechanistic insights into NMDA receptor expression and oxidative stress balance

**DOI:** 10.1038/s41598-024-65301-z

**Published:** 2024-06-20

**Authors:** Nastran Kabiri-Samani, Hossein Amini-Khoei, Mohammad Rahimi-Madiseh, Antoni Sureda, Zahra Lorigooini

**Affiliations:** 1 Student Research Committee, Shahrekord University of Medical Sciences, Shahrekord, Iran; 2 Medical Plants Research Center, Basic Health Sciences Institute, Shahrekord University of Medical Sciences, Shahrekord, Iran; 3 Research Group on Community Nutrition & Oxidative Stress, University of the Balearic Islands-IUNICS, 07122 Palma de Mallorca, Spain; 4 CIBEROBN (Physiopathology of Obesity and Nutrition), Instituto de Salud Carlos III, 28029 Madrid, Spain; 5 Health Research Institute of Balearic Islands (IdISBa), 07120 Palma, Balearic Islands Spain

**Keywords:** Trigonelline, NMDA, Oxidative stress, Seizure, Natural product, Medicinal plant, Diseases of the nervous system, Molecular neuroscience, Diseases, Neurology

## Abstract

Glutamatergic neurotransmission and oxidative stress are involved in the pathophysiology of seizures. Some anticonvulsants exert their effects through modulation of these pathways. Trigonelline (TRG) has been shown to possess various pharmacological effects like neuroprotection. Therefore, this study was performed to determine TRG’s anticonvulsant effects, focusing on its potential effects on N-methyl-D-aspartate (NMDA) receptors, a type of glutamate receptor, and oxidative stress state in the prefrontal cortex (PFC) in PTZ-induced seizure in mice. Seventy-two male mice were randomly divided into nine groups. The groups included mice that received normal saline, TRG at doses of 10, 50, and 100 mg/kg, diazepam, NMDA (an agonist), ketamine (an antagonist), the effective dose of TRG with NMDA, as well as sub-effective dose of TRG with ketamine, respectively. All agents were administrated intraperitoneally 60 min before induction of seizures by PTZ. Latency to seizure, total antioxidant capacity (TAC), and malondialdehyde (MDA) levels in serum and PFC were measured. Furthermore, the gene expression of NR2A and NR2B, subunits of NMDA receptors, was measured in the PFC. TRG administration increased the latency to seizure onset and enhanced TAC while reducing MDA levels in both the PFC and serum. TRG also decreased the gene expression of NR2B in the PFC. Unexpectedly, the findings revealed that the concurrent administration of ketamine amplified, whereas NMDA mitigated, the impact of TRG on latency to seizure. Furthermore, NMDA diminished the positive effects of TRG on antioxidant capacity and oxidative stress, while ketamine amplified these beneficial effects, indicating a complex interaction between TRG and NMDA receptor modulation. In the gene expression of NMDA receptors, results showed that ketamine significantly decreased the gene expression of NR2B when co-administrated with a sub-effective dose of TRG. It was found that, at least partially, the anticonvulsant effect of TRG in PTZ-induced seizures in male mice was mediated by the attenuation of glutamatergic neurotransmission as well as the reduction of oxidative stress.

## Introduction

Epilepsy, characterized as a chronic noncommunicable brain disorder, manifests across all age groups and stands as one of the primary neurological conditions worldwide, impacting nearly 1% of the global populace. Marked by recurrent unprovoked seizures, epilepsy represents the most prevalent chronic neurological ailment, affecting millions globally, with a prevalence spanning from 0.5 to 1% across diverse populations. Given the multifaceted responsibilities of the brain, seizures may disrupt various functions, leading to a multitude of symptoms^1–4^. The pathology of seizures includes various aspects of imbalance in the excitability and inhibition of cerebral cortex activities, impaired neurotransmitter secretion, structural disorders, ion channel function, endogenous neuropeptide depletion, and metabolic diseases of the brain^5^. Findings from recent studies suggest that oxidative stress also plays a pivotal role in the pathogenesis of epileptic seizures^6^.

Studies have reported that local or systemic injections of glutamate, aspartate, stimulant endogenous compounds such as quinoline, some sulfur-containing amino acids, *N*-methyl-d-aspartate (NMDA), and α-amino-3-hydroxy-5-methyl-4-isoxazolepropionic acid (AMPA) receptor agonists, and kinates can induce seizures in animal models. It has also been shown that all types of NMDA antagonists (NMDA competitive antagonists, glycine site antagonists, and calcium channel antagonists), as well as AMPA antagonists and kinates, have broad anticonvulsant properties in acute and chronic models of animal seizures^7^. Accumulation of Ca^2+^ within neurons following activation of NMDA receptors appears to be the leading cause of stimulation in the brain. Due to the critical role of NMDA receptors in brain excitation, NMDA receptor blockade can be considered an effective treatment for seizures^8,9^.

In recent years, the discovery of medical compounds with neuroprotective effects that can prevent the progression and development of the disease has been considered. Studies have shown that some herbs and their active ingredients are able to significantly reduce the severity and duration of seizures through several neuroprotective activities, including antioxidants, anti-inflammatory agents, and alteration of neurotransmitter levels, as well as the expression of their receptors^10,11^.

Trigonelline (TRG) is a herbal bioactive alkaloid (*N*-methyl derivative of niacin) with anti-inflammatory, antioxidant, and neuroprotective activities^12,13^. TRG is found in many plants mainly from the families Lamiaceae, Asteraceae, and Fabaceae, and especially in the seeds of plant species of coffee and fenugreek^12^. Because no study has been performed to evaluate the anticonvulsant effects of TRG, and due to the role of NMDA receptors as well as oxidative stress in the pathophysiology of seizures, this study was conducted to determine the anticonvulsant impact of TRG focusing on its potential effects on NMDA receptor expression and oxidative stress states in the PFC in the pentylenetetrazole (PTZ)-induced seizure in male mice.

## Methods and materials

This study is reported in accordance with ARRIVE guidelines.

### Animals

All the procedures involving NMRI mice in this experiment were conducted following the standards set by Shahrekord University of Medical Sciences and the NIH Guide for the Care and Use of Laboratory Animals (8th edition, National Academies Press). Full efforts were made to reduce the use of animals and to advance their welfare. All the study procedures are approved by “Shahrekord University of Medical Sciences ethics committee” (Ethics committee reference: IR.SKUMS.REC.1397.289).

This study was performed on 72 male NMRI mice aged 8–12 weeks, weighing 20–30 g. The mice were purchased from the Pasteur Institute of Iran (Tehran, Iran). Animals were kept under standard environmental conditions, including a 12-h light/dark cycle, temperature of 23 ± 1 °C, and free access to food and water^14^. Each animal was tested only once.

### Study design

The animals were randomly distributed into nine experimental groups, each consisting of 8 individuals: normal saline (1 ml/kg), TRG at doses of 10, 50, and 100 mg/kg^15^, diazepam (10 mg/kg)^16^, 75 mg/kg of NMDA as an agonist^17^, 0.5 mg/kg of NMDA antagonist (Ketamine)^18^, an effective dose of TRG (100 mg/kg) with NMDA agonist and a sub-effective dose of TRG (10 mg/kg) with ketamine, respectively.

To induce seizures, PTZ was intravenously injected at a dose of 90 mg/kg using an infusion pump at a rate of 1 ml/min. All agents were injected intraperitoneally 60 min before the administration of PTZ^19^. Subsequently, the latency to seizure was recorded in each experimental group. Following this, a blood sample was collected under deep anesthesia induced by ketamine and xylazine, with doses of 60 and 20 mg/kg, respectively. Followed by centrifugation at 3800 rpm for 5 min, serum was isolated^12^. Then, the PFC was isolated from the brain, frozen, and kept at – 70 ℃ until molecular and biochemical evaluations.

### Determination of seizure onset time

A 30-gauge needle was fixed in the mice tail vein for seizure induction. Mice were allowed to move freely. A 5% solution of PTZ at a dose of 90 mg/kg and 1 ml/min rate was continuously injected into the tail vein using a Syringe pump (New 1000, New Era Pump System, Inc.). The injection was stopped as soon as anterior clonus was observed. The onset time of limb contraction from the start of injection of the minimum dose of PTZ (based on mg/kg body weight) to seizure induction is considered an indicator of the threshold for colonic epilepsy^20^.

### Measurement of antioxidant capacity of serum and PFC

The Ferric Reducing Antioxidant Power (FRAP) assay was used to determine the antioxidant capacity of the serum and PFC of the brain. This method (Benzie et al.^21^) was performed using an automatic plate reader (LQ-300+II-Epson, USA) at 37 °C and pH = 3.6 and is based on the ability to convert Fe^3+^ to Fe^2+^ and produce a blue Fe^2+^ complex (TPTZ) with a maximum light absorption of 593 nm.

### Measurement of serum and PFCs malondialdehyde level

Malondialdehyde (MDA) was measured in serum and PFC based on previous described method^17^. The MDA concentration was determined monitoring the formation of color chromophores following the reaction of thiobarbituric acid (TBA) with MDA. Absorbance was recorded for cortex samples at 562 nm and serum samples at 532 nm using an automatic plate reader (LQ-300+II-Epson, USA).

### Measurement of NMDA receptor gene expression in the PFC

The gene expression of NMDA receptor subunits (NR2A, NR2B) was evaluated by real-time PCR. Initially, the RNA of the samples was extracted using Sinagene’s RNX. In the next step, cDNA is synthesized from RNA samples using the Thermofisher kit and following the manufacturer-provided procedure. The specific primers were designed using Vector ATI software (Table 1). Target cDNAs were amplified using a light cycler device (Roche Diagnostics, Mannheim, Germany) for 40 cycles following an initial denaturation step of 10 min at 95 °C. Table 2 lists the conditions used for amplification. The B2m gene was considered a normalizer, and the expression change of our genes was compared with that of the control group. Finally, the changes in the expression of each mRNA were calculated using the 2^− ΔΔCt^ relative expression formula as described previously^22^.

**Table 1:** Sequence of primers.

Gene name	Forward primer (5′–3′)	Reverse primer (5′–3′)
B2m	GGAAGTTGGGCTTCCCATTCT	CGTGATCTTTCTGGTGCTTGTC
NR2A	CTCAGCATTGTCACCTTGGA	GCAGCACTTCTTCACATTCAT
NR2B	CTACTGCTGGCTGCTGGTGA	GACTGGAGAATGGAGACGGC

**Table 2:** Temperature of real time-PCR reactions.

Gene name	Hold (°C-min)	Cycling		Melt (°C–°C)
		(°C-s)	Number of cycles	
B2m	95–10	95–10 56–15 72–20	40	72–95
NR2A	95–10	95–10 62–15 72–20	40	72–95
NR2B	95–10	95–10 62–15 72–20	40	72–95

### Statistical analysis

Data were entered into PRISM statistical software version 8, and the normality of data was evaluated using the Shapiro–Wilk test. Then, data was analyzed through one-way ANOVA followed by the Tukey post hoc test to determine the groups involved in the statistical differences. Results were reported as Mean ± SD, and P < 0.05 was considered statistically significant.

### Ethical approval

All methods were performed according to the relevant guideline and regulations. All procedures were carried out under the regulations of the Shahrekord University of Medical Sciences and the Guide for the Care and Use of Laboratory Animals of the National Institutes of Health and Guide for the Care and Use of Laboratory Animals (8th edition, National Academies Press). All the study procedures are approved by “Shahrekord University of Medical Sciences ethics committee” (Ethics committee reference: IR.SKUMS.REC.1397.289).

## Results

### Effect of TRG on seizure onset time

**Figure 1:**
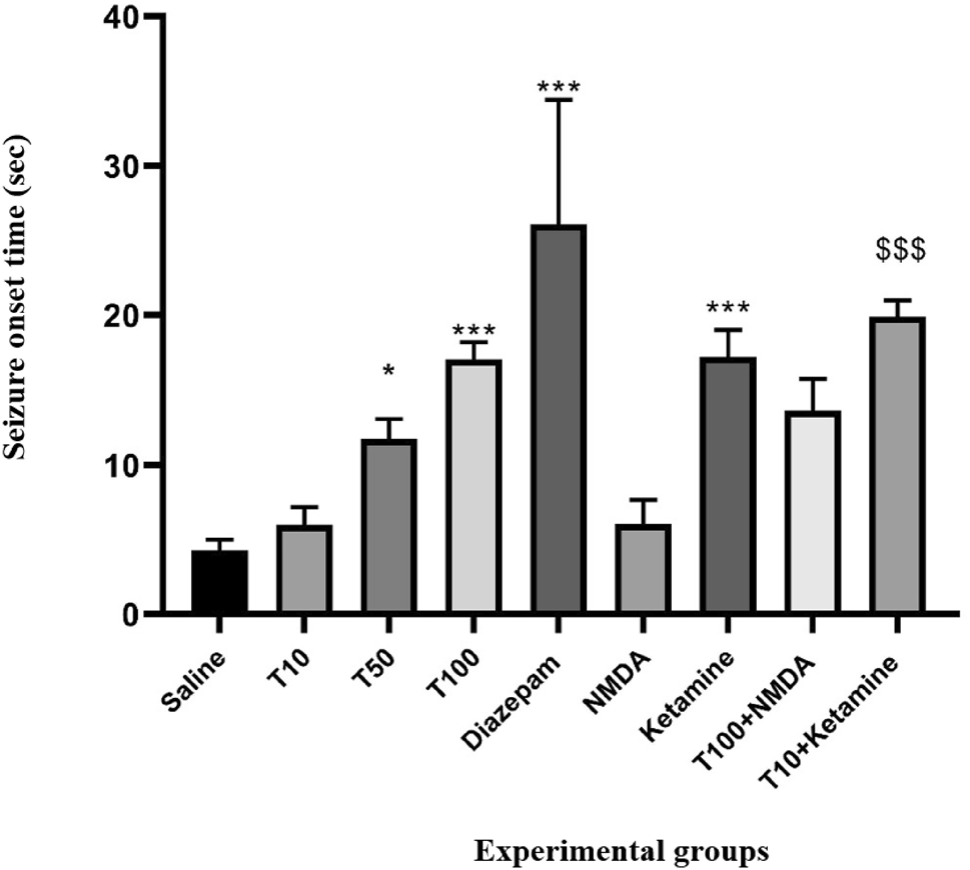
The average seizure onset time in the experimental groups. The results were expressed as Mean ± SD based on the data of 8 samples and were analyzed by one-way ANOVA and Tukey post hoc test. ** Significant difference with the saline-received group at the level of P < 0.001, * significant difference with the saline-received group at the level of P < 0.05, ^$$$^ significant difference with trigonelline 10 at the level of P < 0.001, saline: control group, T: trigonelline.

Based on the results of ANOVA analysis and Tukey post hoc test (Fig. 1), the latency to seizure in the groups that received TRG was higher than in the group that received saline. This increase was significant in the groups that received TRG at 50 and 100 mg/kg (P < 0.05 and P < 0.001, respectively). The average seizure onset time in the Diazpam group was significantly higher than in the saline group (P < 0.001). This time was significantly increased in the group that received ketamine compared to the saline group (P < 0.001) and in the group that received ketamine plus an ineffective dose of TRG compared to the group that received TRG at the dose of 10 mg/kg alone (P < 0.001). The average seizure onset time was insignificant in the group that received NMDA compared to the saline group (P > 0.05). This time was decreased in the group that received NMDA with an effective dose of TRG compared to the group that received TRG at the dose of 100 mg/kg alone. This reduction was not significant (P > 0.05).

### Effect of TRG on serum total antioxidant capacity

**Figure 2:**
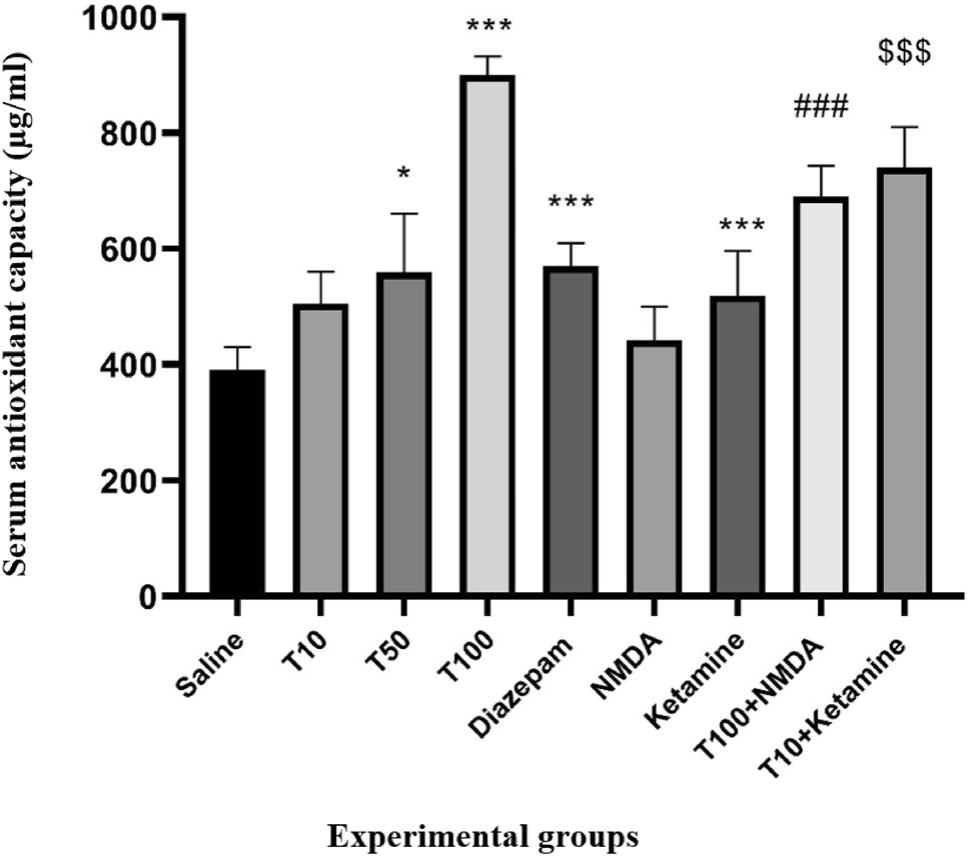
Serum antioxidant capacity in the experimental groups. The results were expressed as Mean ± SD from the mean based on the data of 8 samples and were analyzed by one-way ANOVA and Tukey post hoc test. ***Significant difference with the saline-received group at the level of P < 0.001, * significant difference with the saline-received group at the level of P < 0.05, ### significant difference with trigonelline 100 at the level of P < 0.001, $$$ significant difference with trigonelline 10 at the level of P < 0.001. Saline: control group, T: trigonelline.

Serum total antioxidant capacity increased in the groups that received TRG compared to the saline-received group (Fig. 2). This increase was significant in the groups that received TRG at 50 and 100 mg/kg (P < 0.05 and P < 0.001, respectively). Additionally, this rate was significantly lower in the group of NMDA plus the effective dose of TRG than in the group of TRG 100 (P < 0.001). In contrast, in the group that received ketamine plus an ineffective dose of TRG (10 mg/ml), the response was more significant than the group of TRG 10 mg/kg alone (P < 0.001).

### Effect of TRG on PFC total antioxidant capacity

**Figure 3:**
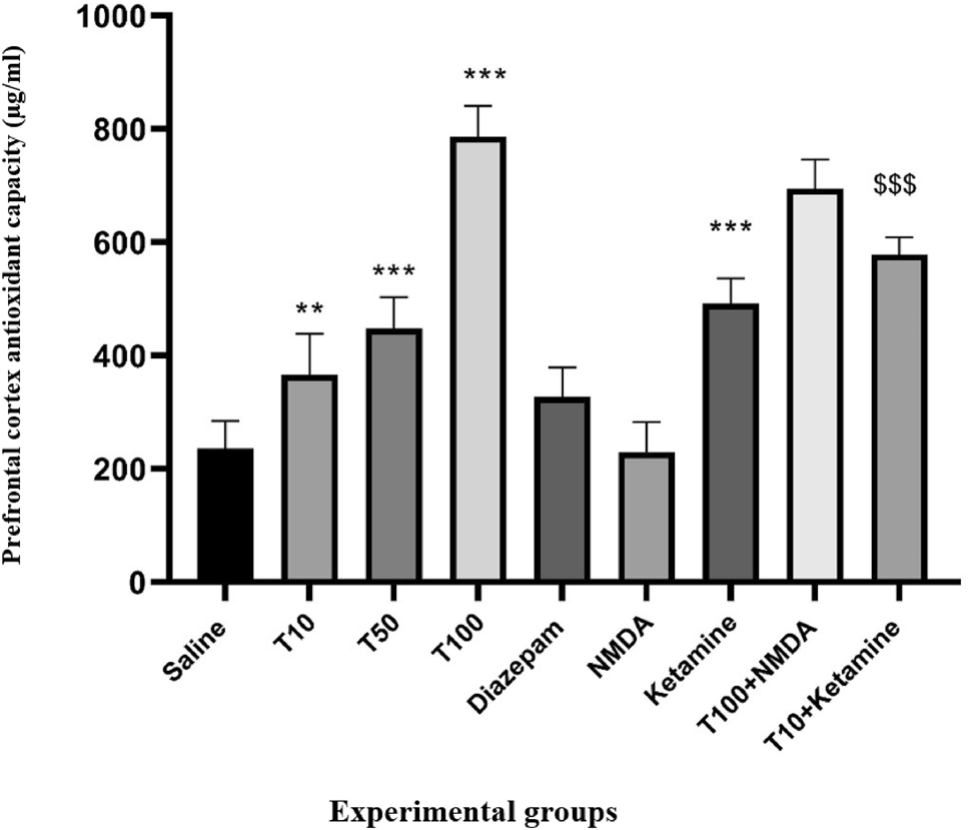
Prefrontal cortex antioxidant capacity in the experimental groups. The results were expressed as Mean ± SD from the mean based on the data of 8 samples and were analyzed by one-way ANOVA and Tukey post hoc test. ***Significant difference with the saline-received group at the level of P < 0.001, ** Significant difference with the saline-received group at the level of P < 0.01, ^$$$^ significant difference with trigonelline 10 at the level of P < 0.001, saline: control group, T: trigonelline.

The total antioxidant capacity of the PFC in mice was higher in the groups receiving TRG at doses of 10, 50, and 100 mg/kg than in the saline-received group (P < 0.01, P < 0.001, and P < 0.00, respectively). The increase in antioxidant capacity was particularly pronounced in the TRG 100 group. Furthermore, this increase was significantly higher in the groups that received ketamine than the saline-received group (P < 0.001). Notably, the increase in antioxidant capacity was significant in the ketamine group with TRG 10 compared to the group with TRG 10 alone (P < 0.001) (Fig. 3).

### Effect of TRG on serum MDA levels

**Figure 4:**
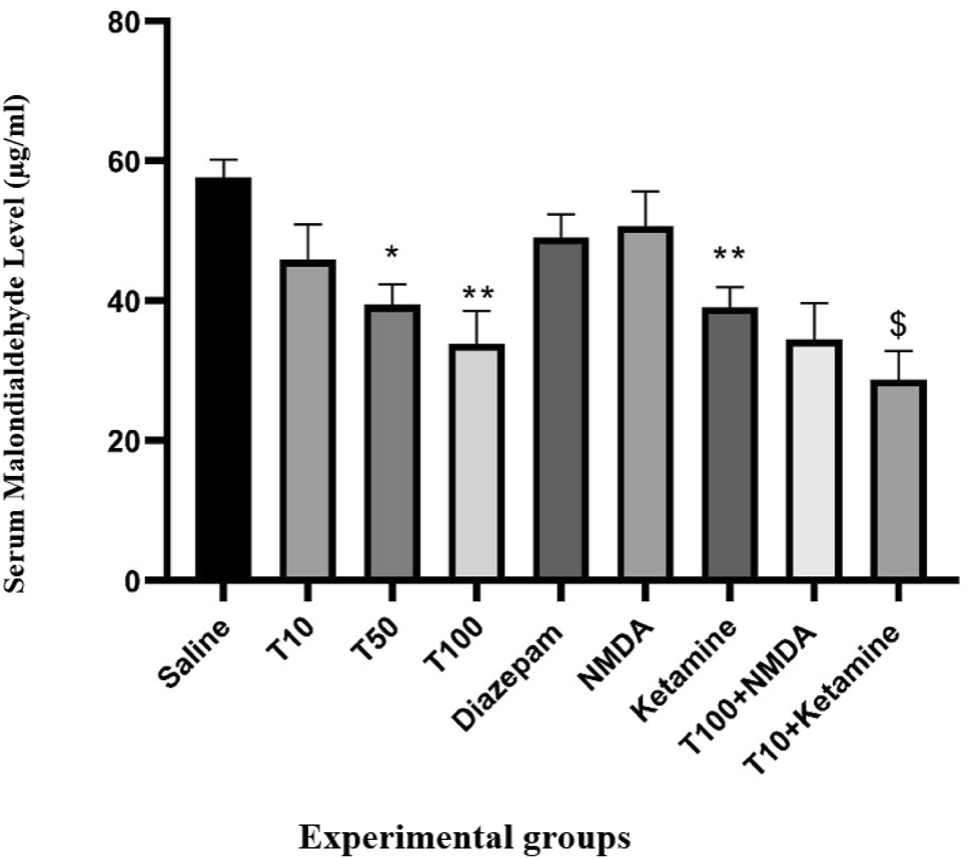
Serum malondialdehyde levels in the experimental groups. The results were expressed as Mean ± SD from the mean based on the data of 8 samples and were analyzed by one-way ANOVA and Tukey post hoc test. **Significant difference with the saline-received group at the level of P < 0.01, ^$^ Significant difference with trigonelline 10 at the level of P < 0.05, saline: control group, T: trigonelline.

Serum MDA levels were lower in all three groups receiving TRG than in the saline-received group, with significance observed in the TRG 50 and 100 mg/kg groups (P < 0.05 and P < 0.01, respectively). Additionally, MDA levels in the ketamine were significantly lower than those in the saline-received group (P < 0.01). Moreover, serum MDA levels in the ketamine group with TRG 10 were notably lower than in the group with TRG 10 mg/kg (P < 0.05) (Fig. 4).

### Effect of TRG on PFC MDA levels

**Figure 5:**
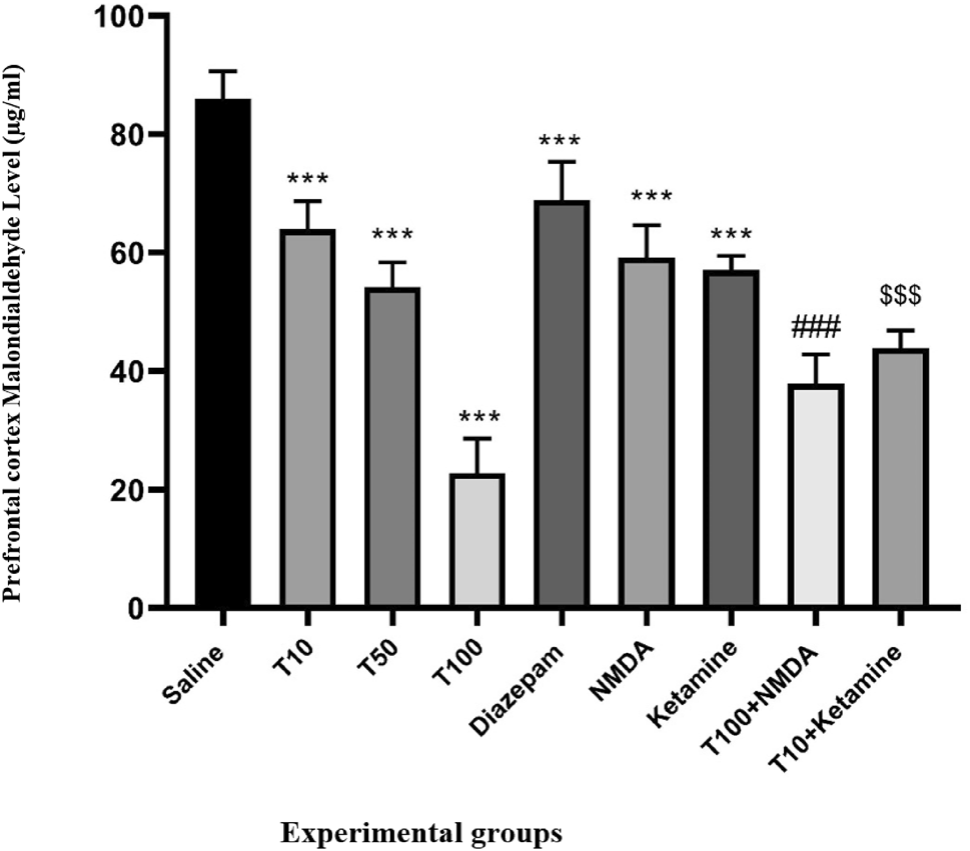
Prefrontal cortex malondialdehyde level in the experimental groups. The results were expressed as Mean ± SD from the mean based on the data of 8 samples and were analyzed by one-way ANOVA and Tukey post hoc test. ***Significant difference with saline-received group at level P < 0.001, ### Significant difference with trigonelline 100 at level P < 0.001, $$$ significant difference with trigonelline 10 at level P < 0.001. Saline: control group, T: trigonelline.

MDA levels in PFC in the saline-received group was significantly higher than in the groups that received TRG (P < 0.001) (Fig. 5). Additionally, diazepam, NMDA, and ketamine significantly reduced the MDA level in the PFC compared to the saline-received group (P < 0.001). Moreover, MDA levels in the group that received NMDA with TRG 100 were significantly higher than in the TRG 100 group alone (P < 0.001). Similarly, in the group that received ketamine with TRG 10, the PFC MDA levels were substantially lower than in the TRG 10 group alone (P < 0.001).

### Effect of TRG on NMDA receptor gene expression

**Figure 6:**
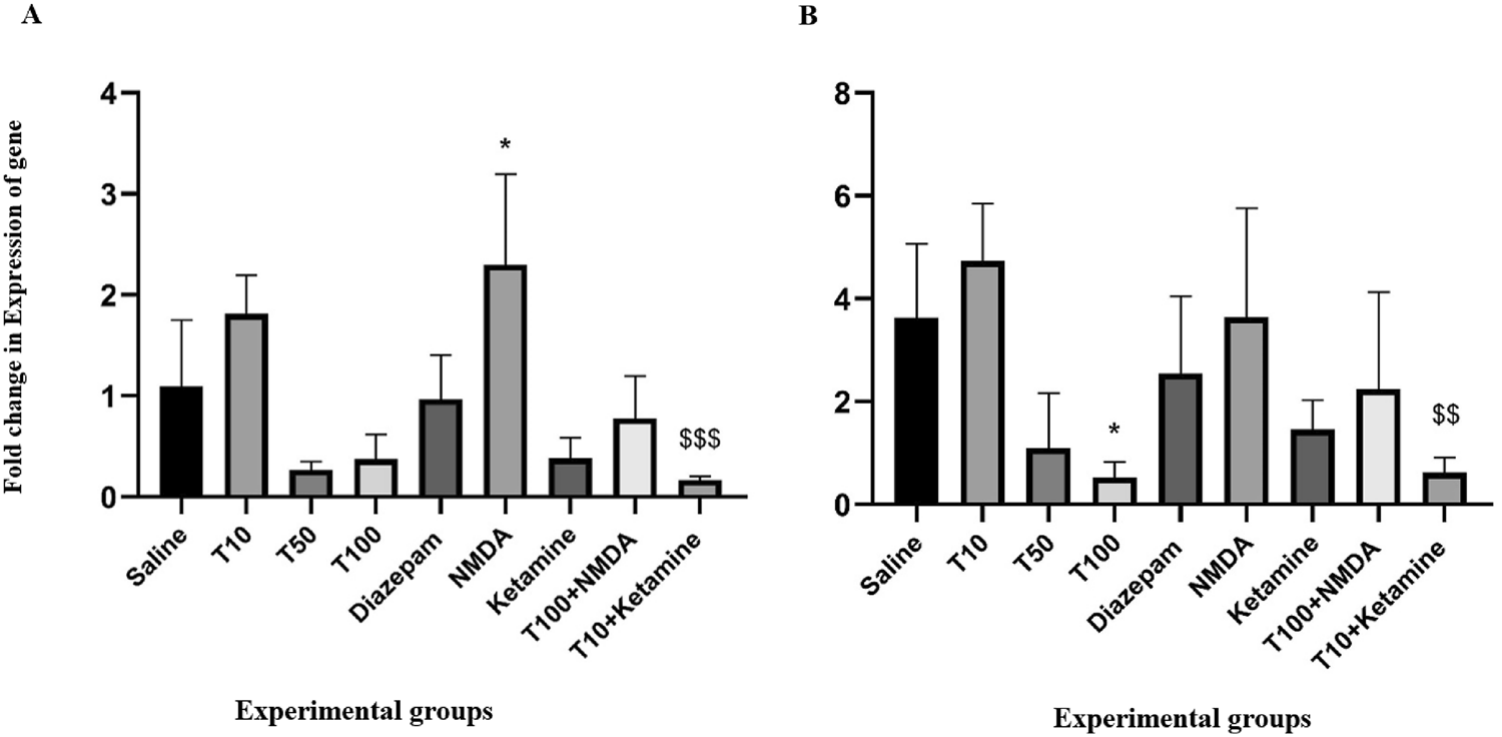
(**A**) Expression of NR2A receptor gene in the experimental groups. The results were expressed as Mean ± SD from the mean based on the data of 8 samples and were analyzed by one-way ANOVA and Tukey post hoc test. ^*^ Significant difference with saline-received group at the level of P < 0.05, ^$$$^Significant difference with trigonline 10 at the level of P < 0.001. (**B**) Expression of NR2B receptor gene in the experimental groups. ^*^Indicates a significant difference with the saline-received group at P < 0.05, ^$$^ significant difference with trigonelline 10 at the level of P < 0.01, saline: control group, T: trigonelline.

According to the results of Fig. 6, the expression of the NR2A receptor subunit in the PFC was not significantly different between the TRG 50 and 100 mg/kg-received groups and the saline-received group (P > 0.05). However, the expression of the NR2B subunit was reduced considerably in the TRG 100 group compared to the saline-received group (P < 0.05). Furthermore, NMDA significantly increased NR2A gene expression compared to the saline-received group (P < 0.05). Additionally, in the group that received ketamine with TRG 10, the expression of both NR2A and NR2B genes was significantly reduced compared to the TRG 10 mg/kg group alone (P < 0.001 and P < 0.01, respectively).

## Discussion

Based on the results of the present study, it is evident that TRG, especially at higher doses (100 mg/kg), can enhance the tolerance to seizures induced by PTZ. Moreover, TRG-exposed groups exhibited increased antioxidant capacity, reduced MDA levels in both serum and PFC and decreased expression of NMDA receptors in the PFC.

TRG is a pyridine alkaloid plant hormone synthesized by the methylation of nicotinic acid that regulates plant growth and survival^23^. It is in significant quantities in many plants, including coffee beans, fenugreek seeds, and tung-kua-jen^24^. Previous research has identified pharmacological activities associated with TRG, including hypoglycemic, hypolipidemic, neuroprotective, anti-migraine, sedative, and memory-enhancing effects^15,25–27^. TRG has also been reported to exert neuroprotective properties in rodent models of Parkinson’s disease^28^. Additionally, TRG has shown promise in alleviating symptoms of depression and anxiety^12^.

Given the global prevalence of epilepsy and the limitations of current treatment options with significant side effects, there is a growing interest in exploring alternative agents such as TRG for epilepsy management^29,30^. Numerous studies have demonstrated the neuroprotective properties of TRG and its ability to reduce the severity and duration of epileptic seizures in various models of epilepsy^31^. In this sense, pre-treatment with TRG has been shown to improve the motor symptoms of mice with Parkinson’s disease. This effect has been attributed to the neuroprotective properties of TRG which is associated with an attenuation of neuronal damage and inflammation while preventing apoptosis^15,19^. Tohda et al.^32^ showed that TRG regenerates neural networks, improves memory, and potentially slows the progression of neurodegenerative diseases. Therefore, it is possible that this compound, with its neuroprotective effects, can prevent nerve damage and, consequently, convulsive stimuli. In fact, oxidative stress can alter neurotransmission, neuronal function, and brain activity^33^. Oxidative stress has also been suggested as an independent factor in neuronal cell death that can play an essential role in neural diseases such as seizures^34^.

Consistent with previous studies, the present research found that TRG administration increased seizure onset time, particularly at 50 and 100 mg/kg doses. Furthermore, the antioxidant capacity was significantly higher in the TRG groups compared to the saline-received group, with the highest increase observed in the TRG 100 group. In accordance with a previous study using kainic acid for inducing temporal lobe epilepsy, the administration of TRG significantly increased the mean seizure onset time in the TRG-received groups compared to the saline-received group^13^. This increase was statistically significant in the TRG groups at 50 and 100 mg/kg doses. In addition, the antioxidant capacity in the TRG groups was higher than in the saline-received group, whereas the pro-inflammatory mediators were decreased. In the study by Zhou et al.^35^, using streptozotocin-induced diabetic rats, TRG decreased oxidative stress induced by diabetes. Also, in a study conducted in an experimental model of diabetes, TRG significantly reduced oxidative stress markers by reducing lipid peroxidation, increasing antioxidant capacity, and increasing the activity of antioxidant enzymes in various tissues such as the liver, kidney, and serum^36^. Altogether, it suggests that this natural compound with high antioxidant potential seems effective in many oxidative stress-related diseases, including seizures.

TRG appears to be able to exert its antioxidant properties with its structural basis and functional groups. It is a natural alkaloid characterized by a pyridine ring, which stabilizes free radicals by transferring electrons, thereby reducing oxidative stress. The N-methylniacin derivative in TRG increases lipophilicity, enhances interaction with lipid membranes, and protects them from lipid peroxidation. The carboxylate group in TRG donates hydrogen atoms to neutralize free radicals and overall increases its antioxidant activity^37^. This antioxidant effect is essential in reducing neuronal excitability and seizure susceptibility, highlighting the therapeutic potential of TRG in epilepsy and other neurodegenerative conditions.

Animal studies have suggested that PTZ-induced seizures may involve increased expression of NR2B subunits of NMDA receptors in the hippocampus since the selective blockade of NR2B subunits by ifenprodil significantly attenuated seizures^38^. Human studies have also reported an increase in the expression of NR2A and NR2B subunits in epilepsy associated with focal cortical dysplasia^39^. In a survey by Mathern et al. on patients with temporal lobe seizures, an increase in mRNA levels of ionotropic glutamate receptors and a change in the composition of ionotropic glutamate receptor subunits were reported, and it was suggested that these changes may be involved in neuronal stimulation, neuronal synchronization, and seizures^40^. According to the results of the present study, TRG administration led to a significant reduction in the expression of the NR2B subunit in the PFC, while the decrease in NR2A expression was not statistically significant. This inhibition of the expression of these subunits could contribute to the delay in the onset of seizures. The observed differential response of TRG on NR2B and NR2A subunits requires further investigation. This raises the question of whether this response, in parallel with many drugs, is due to its selective action and increased affinity for the NR2B subunit. Previous studies have reported drugs that can selectively inhibit the NR2B subunit^41–44^. However, elucidating the mechanisms underlying TRG's distinct effects on NMDA receptor subunits requires comprehensive exploration and potentially paves the way for new therapeutic interventions targeting specific subunits.

Given the pivotal role of NMDA receptors in neuronal stimulation and epilepsy, NMDA receptor blockade has emerged as a promising therapeutic approach to treat seizures^9,45^. In this sense, there has been an increased expression of NMDA receptor subunits following epilepsy^46^. In the present study, ketamine, an NMDA receptor blocker, significantly increased seizure onset time and antioxidant capacity while reducing MDA levels in serum and PFC tissue, indicating the role of NMDA receptors in seizures. Furthermore, co-administration of NMDA with TRG 100 (effective dose) significantly decreased the serum antioxidant capacity and increased MDA level compared of PFC to the group that received TRG 100 alone. Also, the injection of ketamine with TRG 10 (sub-effective dose) significantly increased the duration of seizure delay and antioxidant capacity of serum and PFC and decreased serum and PFC MDA levels compared to the group that received TRG 10. These findings highlight that the anticonvulsant effect of TRG may be mediated through its mitigating effects on NMDA receptors, which consequently attenuated oxidative stress and exerted an anticonvulsant effect.

In support of this, docking studies show that TRG can effectively target the NMDA receptor and form stable interactions through stacking and hydrogen bonding, although the binding affinity (score − 1.73) is lower than that of the inhibitor. Native DK1 (− 2.96) is less.

In summary, the interaction of TRG with the NMDA receptor is significant, involving key residues that are critical for receptor function. This interaction suggests the potential therapeutic effects of TRG through modulation of the NMDA receptor, although its binding affinity is somewhat lower compared to other inhibitors.

In summary, trigonelline demonstrates the strongest binding affinity and therapeutic potential with the Insulin Receptor Tyrosine Kinase, followed by NMDAR, AMPAR, α7 nAChR, 5HT1AR, and the muscarinic acetylcholine receptors, suggesting a multifaceted mechanism through which it can exert its anticonvulsant effects^47^. Further, in vivo studies are necessary to validate these findings and fully understand the clinical relevance of TRG's receptor interactions.

According to previous studies, LD_50_ of TRG was determined to be 5000 mg/kg, a concentration much higher than the dose used in this research, which does not imply toxicity for the mice analyzed^25,48^. The non-toxicity of this compound in the dose used in this study is a strong point in its application in future clinical studies.

## Conclusion

Based on the results of the present study, it is evident that TRG, especially at doses of 100 mg/kg, can enhance tolerance to seizures induced by PTZ. Additionally, TRG-exposed groups exhibited increased antioxidant capacity, reduced MDA levels in both serum and PFC, and decreased expression of NMDA receptors in the PFC. The anticonvulsant effect of TRG in PTZ-induced seizures in male mice was mediated, at least partially, by the attenuation of glutamatergic neurotransmission as well as the reduction of oxidative stress. Overall, the findings of this study provide valuable insights into the neuroprotective and anticonvulsant properties of TRG, suggesting its potential as a therapeutic agent for epilepsy and other neurological disorders. Future research could focus on elucidating the precise mechanisms behind TRG’s anticonvulsant effects evaluating its efficacy and safety in clinical trials.

## Data Availability

At the Medical Plants Research Center, Shahrekord University of Medical Sciences, data concerning the current study can be obtained. The datasets generated and/or analysed during the current study are available in the Zahra Lorigooini repository, [z.lorigooini@gmail.com].

## Abbreviations

TRG: Trigonelline
PTZ: Pentylenetetrazole
TAC: Ttotal antioxidant capacity
MDA: Malondialdehyde
NMDA: *N*-Methyl-d-aspartate
PFC: Prefrontal cortex
AMPA: α-Amino-3-hydroxy-5-methyl-4-isoxazolepropionic acid
Fe3+-TPTZ: Ferric-tripiridyltriazine
TBA: Thiobarbituric acid
